# Association of hypocalcemia with in-hospital mortality in critically ill patients with intracerebral hemorrhage: A retrospective cohort study

**DOI:** 10.3389/fneur.2022.1054098

**Published:** 2023-01-09

**Authors:** Fang Gu, Wenyan Zhao, Xiangjie Duan, Ying Zhang, Xiaoming Luo, Guoqing Chen, Xiaoli Jin, Hangli Pan, Faliang Gao, Huadong Wu

**Affiliations:** ^1^Center for Reproductive Medicine, Department of Pediatrics, Zhejiang Provincial People's Hospital, Affiliated People's Hospital, Hangzhou Medical College, Hangzhou, Zhejiang, China; ^2^Center for General Practice Medicine, Department of General Practice Medicine, Zhejiang Provincial People's Hospital, Affiliated People's Hospital, Hangzhou Medical College, Hangzhou, Zhejiang, China; ^3^Department of Infectious Diseases, The First People's Hospital of Changde, Changde, Hunan, China; ^4^Department of Pediatrics, Hangzhou TCM Hospital Affiliated to Zhejiang Chinese Medical University, Hangzhou, Zhejiang, China; ^5^Center for Rehabilitation Medicine, Department of Neurosurgery, Zhejiang Provincial People's Hospital, Affiliated People's Hospital, Hangzhou Medical College, Hangzhou, Zhejiang, China; ^6^Key Laboratory of Endocrine Gland Diseases of Zhejiang Province, Hangzhou, Zhejiang, China; ^7^Center for Rehabilitation Medicine, Department of Neurology, Zhejiang Provincial People's Hospital, Affiliated People's Hospital, Hangzhou Medical College, Hangzhou, Zhejiang, China

**Keywords:** hypocalcemia, mortality, intracerebral hemorrhage, intensive care unit, hospital

## Abstract

**Background and purpose:**

There was little evidence to study the relationship between hypocalcemia and mortality among critically ill patients with intracerebral hemorrhage (ICH) aged ≥16 years. This study aimed to determine the potential association between hypocalcemia and in-hospital and ICU mortality in patients with ICH in the United States.

**Methods:**

We analyzed 1,954 patients with ICH from the e-Intensive Care Unit Collaborative Research Database and divided them into hypocalcemia and non-hypocalcemia groups. Hypocalcemia was defined as albumin-adjusted total calcium below 8.4 mg/dl. The primary and secondary outcomes were hospital and ICU mortality, respectively. We performed multivariable regression and subgroup analyses to evaluate the association of hypocalcemia with hospital and ICU mortality. Cumulative survival rate analysis was performed using Kaplan–Meier curves with log-rank statistics.

**Results:**

We enrolled 1,954 patients with ICH who had been hospitalized in ICU for >24 h and were older than 16 years (average age, 61.8 years; men, 56.7%). We noted that 373 (19%) hospital mortality occurred, including 235 (12%) ICU mortality. In this sample, 195 patients had hypocalcemia. Multivariable logistic regression analyses showed that hypocalcemia was associated with a 67% increased risk of in-hospital and a 72% increased risk of ICU mortality. This association was consistent across subgroup analyses.

**Conclusions:**

Hypocalcemia was associated with a high risk of hospital and ICU mortality among critically ill patients with ICH. Future prospective, randomized, controlled studies are needed to confirm our results.

## 1. Introduction

Intracerebral hemorrhage (ICH) is a life-changing event for patients and their families, and it is a potentially life-threatening medical emergency in hospitalized, critically ill patients (1). ICH has multiple pathophysiological etiologies and is associated with high morbidity and mortality rates (2, 3). However, early identification, accurate diagnosis, and aggressive treatment can improve the chances of recovery in patients with ICH.

Many factors play an important role in biological processes, and calcium (Ca) is such a nutrient for the human body (4). It plays an important role in blood coagulation, blood pressure regulation, platelet function, muscle contraction, hormone regulation, and enzyme activation. Ca plays an essential role in brain injury following ICH by affecting coagulation, regulating blood pressure, and other mechanisms (5, 6). Hypocalcemia is common in pediatric patients and critically ill adults (7–9). It appears to promote coagulopathy and increase blood pressure (10, 11).

Several studies have demonstrated that hypocalcemia is strongly associated to hematoma expansion and worse short-and long-term outcomes in patients with ICH (12–14). Based on these findings, hypocalcemia may be a potential prognostic factor during hospitalization in critically ill patients with ICH; however, studies in this area are lacking. Therefore, we conducted a retrospective cohort study to investigate the association between hypocalcemia and in-hospital and ICU mortalities in critically ill patients with ICH.

## 2. Methods

### 2.1. Data source

All data were drawn from the e-Intensive Care Unit Collaborative Research Database (eICU-CRD), a large multicenter ICU database that includes data on more than 200,000 patients admitted to the ICU at 208 United States hospitals in 2014 and 2015 (15). All data were stored and retrieved electronically and provided by Philips Healthcare in collaboration with the MIT Computational Physiology Laboratory (15). All data were anonymized prior to our analysis using the eICU protocol. Eicu-crd.mit.edu is the web address of this database. The use of this database was approved by PhysioNet review boards. One of the authors (Wenyan Zhao) gained access and was responsible for downloading the data (certification number: 42608104).

### 2.2. Study population

**Figure 1:**
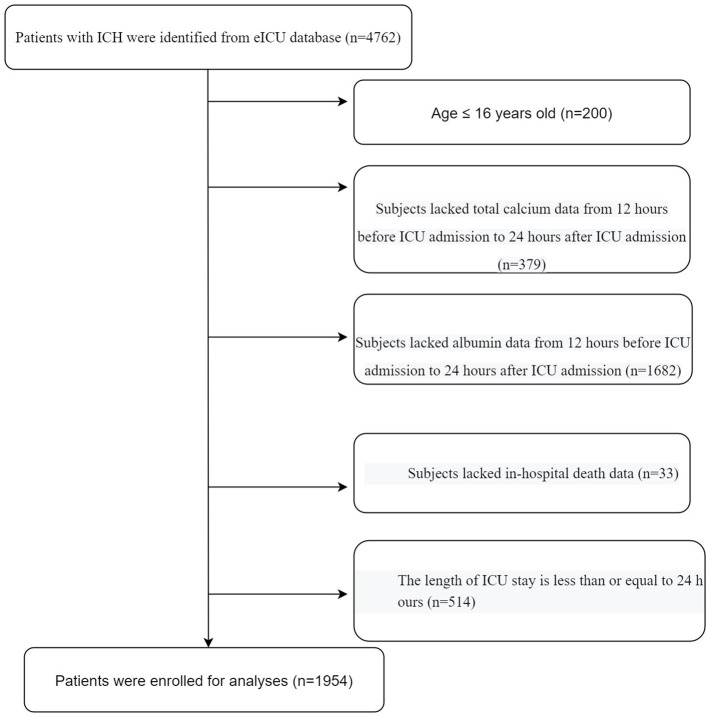
Flow chart of participants.

All patients diagnosed with ICH upon ICU admission were included in this study. The diagnoses of ICH include hemorrhagic stroke, subarachnoid hemorrhage, and intraventricular hemorrhage. The following exclusion criteria were used: (1) age <16 years, (2) total Ca and albumin data missing from 12 h before to 24 h after ICU admission, (3) missing in-hospital death data, and (4) ICU stay ≤24 h. A flowchart of the study is depicted in Figure 1.

### 2.3. Study variables

In this study, hypocalcemia was the exposure variable. The primary and secondary outcomes of our study were in-hospital and ICU mortalities, respectively. All patients were divided into hypocalcemia and non-hypocalcemia groups. Hypocalcemia was defined as an albumin-adjusted total Ca level below 8.4 mg/dl. The albumin-adjusted total Ca level was calculated based on the following formula: albumin-adjusted total Ca (mg/dl) = total Ca (mg/dl) + 0.8 × [4-measured albumin (g/dl)] (16). Total Ca and albumin were first measured from 12 h before to 24 h after ICU admission. Hospital and ICU stay durations were also calculated in this study.

### 2.4. Other variables

Further, we also collected additional data from the eICU-CRD. Sex, age, ethnicity, and region were acquired from the patient and apache patient result tables. Physiological variables, including diastolic blood pressure (DBP), systolic blood pressure (SBP), and body mass index (BMI), were obtained from the Apache Aps Var table. The laboratory indices were platelet (PLT), white blood cell (WBC) count, hemoglobin (Hb), glutamic pyruvic transaminase (GPT), prothrombin time (PT), creatinine (Cr), international normalized ratio (INR), blood urea nitrogen (BUN), magnesium (Mg), glucose (GLU), and lactate. Comorbidities, including coronary artery disease, hypertension, atrial fibrillation, diabetes, and congestive heart failure, were extracted from the APACHE IV score. Patients with ICH, including those diagnosed with hemorrhagic stroke, subarachnoid hemorrhage, and intraventricular hemorrhage, were extracted from the diagnosis table. The severity of ICU admission was evaluated by using the sequential organ failure assessment (SOFA) score. The causes of ICH include traumatic and spontaneous hemorrhage. The information on the use of vasopressor and sedatives on the first day was gathered from Treatment table. All covariates were collected on the first day of ICU admission.

### 2.5. Statistical analysis

Categorical variables were analyzed using percentages, whereas continuous variables were expressed using the means (standard deviation, SD). Firstly, this study used linear regression models and chi-square tests to compare the patients' baseline characteristics and outcomes in different groups. Secondly, we calculated three multivariable logistic regression models simultaneously as follows: model 1, not adjusted; model 2, adjusted for sex, age, and race/ethnicity; model 3, adjusted for model 1+ region, causes of ICH, BMI, SBP, WBC, PLT, Hb, GPT, INR, PT, GLU, BUN, Cr, Mg, lactate, SOFA score, first-day vasopressor, first-day sedative, atrial fibrillation, and congestive heart failure. The covariates selected for adjustment were based on the fact that the addition of covariates to the model changed the regression coefficient by at least 10%. 95% confidence interval (CI) and odds ratios (OR) were estimated for all the models. Thirdly, we performed stratified analyses and interactions in the light of age, sex, region, causes of ICH, SOFA score, histories of hypertension, diabetes, and BMI. Finally, Kaplan–Meier curves were constructed for primary and secondary outcomes.

To confirm the robustness of our results, we quantified unmeasured confounders of hypocalcemia and ICU mortality by calculating *E*-values (17), because unmeasured confounders may affect the observed correlation between hypocalcemia and mortality. The *E*-value allows estimation of the required validity of a confounder.

All tests were two-sided, and *P* < 0.05 was considered statistically significant. All analyses were performed using the R statistical software package (http://www.R-project.org, The R Foundation for Statistical Computing, Vienna, Austria) and Free Statistics software versions 1.5 (18).

## 3. Results

### 3.1. Patient selection

The selection process for the study population was depicted in Figure 1. Firstly, we excluded patients aged ≤16 years (*n* = 200). Secondly, we excluded patients without total Ca (*n* = 379) or albumin (*n* = 1,682) data. Thirdly, we excluded patients without hospital death data (*n* = 33). Fourthly, we excluded patients with an ICU stay time ≤24 h (*n* = 514). Finally, a total of 1,954 eligible patients were enrolled in the analyses.

### 3.2. Baseline characteristics

The selected patients' characteristics were shown in Table 1. Based on the albumin-adjusted total Ca levels, we divided all patients into the hypocalcemia and non-hypocalcemia group. The average age was 61.82 years (men: 56.76%). Among 709 patients with traumatic and 1,245 patients with spontaneous ICH, 108 and 87 patients developed hypocalcemia, respectively. Patients with traumatic ICH had a higher incidence of hypocalcemia. Compared with patients without hypocalcemia, those with hypocalcemia had a higher WBC count, GPT, and SOFA score and lower BMI, SBP, PLT, INR, PT, and BUN. In addition, the hypocalcemia group had a lower proportion of patients with histories of chronic disease such as hypertension, diabetes, chronic pulmonary disease, atrial fibrillation, stroke, coronary artery disease, and congestive heart failure. However, there were no differences in age, region, DBP, Hb, Mg, or GLU between the two groups.

**Table 1:** Characteristics of study participants.

**Characteristics**	**Albumin-adjusted total calcium, mg/dl**				***P*-value**
	**Total**	**Non-hypocalcemia** ≥**8.4**	**Hypocalcemia**<**8.4**	
*N*	1,954	1,759	195	
Age, years	61.82 (17.17)	63.11 (16.66)	50.25 (17.47)	< 0.010
**Gender**, ***N*** **(%)**				
Male	1,108 (56.76)	990 (56.31)	118 (60.82)	0.229
**Race/ethnicity**, ***N*** **(%)**				
Caucasian	1,424 (72.88)	1,278 (72.65)	146 (74.87)	< 0.001
African American	225 (11.51)	211 (12.00)	14 (7.18)	
Hispanic	104 (5.32)	96 (5.46)	8 (4.10)	
Asian	56 (2.87)	49 (2.79)	7 (3.59)	
Native American	12 (0.61)	6 (0.34)	6 (3.08)	
Other/unknown	133 (6.81)	119 (6.77)	14 (7.18)	
**Region**, ***N*** **(%)**				
Midwest	654 (33.47)	582 (33.09)	72 (36.92)	0.037
South	463 (23.69)	420 (23.88)	43 (22.05)	
West	535 (27.38)	484 (27.52)	51 (26.15)	
Northeast	169 (8.65)	145 (8.24)	24 (12.31)	
Missing	133 (6.81)	128 (7.28)	5 (2.56)	
**Causes of ICH**, ***N*** **(%)**				
Trauma	709 (36.28)	601 (34.17)	108 (55.38)	< 0.001
Spontaneous	1,245 (63.72)	1,158 (65.83)	87 (44.62)	< 0.001
SOFA score	4.99 (3.36)	4.81 (3.32)	6.62 (3.29)	< 0.001
**Physical examination**				
BMI, kg/m^2^	27.89 (6.76)	28.05 (6.86)	26.43 (5.55)	0.002
SBP, mmHg	131.45 (16.40)	132.02 (16.50)	126.44 (14.63)	< 0.001
DBP, mmHg	70.21 (11.28)	70.17 (11.26)	70.49 (11.46)	0.716
**Laboratory data**				
WBC count, × 10^3^/μl	11.64 (8.78)	11.47 (9.10)	13.17 (4.60)	0.011
Hb, g/ml	12.62 (2.14)	12.64 (2.16)	12.46 (2.00)	0.249
PLT, × 10^3^/μl	211.53 (77.69)	212.75 (78.65)	200.43 (67.47)	0.037
GPT, U/L	39.55 (72.18)	37.54 (69.29)	57.48 (92.36)	< 0.001
BUN, mg/dl	18.98 (13.75)	19.48 (14.06)	14.48 (9.36)	< 0.001
Creatinine, mg/dl	1.17 (1.19)	1.18 (1.18)	1.10 (1.27)	0.371
Magnesium, mg/dl	1.89 (0.30)	1.90 (0.30)	1.85 (0.27)	0.058
Glucose, mg/dl	145.15 (51.61)	145.20 (52.39)	144.69 (44.12)	0.896
Lactate, mmol/L	2.57 (1.95)	2.54 (2.01)	2.77 (1.60)	0.314
INR	1.19 (0.38)	1.19 (0.39)	1.13 (0.20)	0.024
Prothrombin time, s	14.15 (3.85)	14.22 (3.99)	13.48 (2.19)	0.016
First day vasopressor, *N* (%)	111 (5.68)	92 (5.23)	19 (9.74)	0.01
	**Total**	**Non-hypocalcemia** ≥**8.4**	**Hypocalcemia**<**8.4**	
First day sedative, *N* (%)	338 (17.30)	284 (16.15)	54 (27.69)	< 0.001
**Comorbidities**				
Hypertension, *N* (%)	1,024 (52.41)	963 (54.75)	61 (31.28)	< 0.001
Coronary artery disease, *N* (%)	163 (8.34)	156 (8.87)	7 (3.59)	0.011
Atrial fibrillation, *N* (%)	206 (10.54)	204 (11.60)	2 (1.03)	< 0.001
Congestive heart failure, *N* (%)	134 (6.86)	130 (7.39)	4 (2.05)	0.005
Diabetes, *N* (%)	394 (20.16)	378 (21.49)	16 (8.21)	< 0.001
Chronic pulmonary disease, *N* (%)	114 (5.83)	110 (6.25)	4 (2.05)	0.018
Stroke, *N* (%)	263 (13.46)	251 (14.27)	12 (6.15)	0.002

Continuous variables were presented as mean (SD), calculated by linear regression model. Categorical variables were presented as numbers (%), calculated by chi-square test.

SD, standard deviation; BMI, body mass index; SBP, systolic blood pressure; DBP, diastolic blood pressure; WBC, white blood cell; PLT, platelet; INR, international normalized ratio; SOFA, Sequential Organ Failure Assessment; GPT, glutamic-pyruvic transaminase; BUN, blood urea nitrogen.

### 3.3. The outcomes

The primary and secondary outcomes of our study were shown in Table 2. In-hospital mortality was 28.72% in the hypocalcemia group and 18.02% in the non-hypocalcemia group, and the difference was statistically significant (*P* < 0.001). The ICU mortality in the hypocalcemia group was 21.03%, while it was 11.03% in the non-hypocalcemia group (*P* < 0.001). The patients with hypocalcemia had higher in-hospital and ICU mortality than those without hypocalcemia. In the hypocalcemia group, the total length of the hospital stay and ICU stay were 13.33 and 7.91 days, respectively, which were longer than that in the non-hypocalcemia group.

**Table 2:** The outcomes of patients in hypocalcemia and non-hypocalcemia group.

	**Albumin-corrected total calcium, mg/dl**			
	**Total**	**Non-hypocalcemia**	**Hypocalcemia**	***P*** **-value**
		≥**8.4**	<**8.4**	
*N*	1,954	1,759	195	
In-hospital mortality, *N* (%)	373 (19.09)	317 (18.02)	56 (28.72)	< 0.001
ICU mortality, *N* (%)	235 (12.03)	194 (11.03)	41 (21.03)	< 0.001
Hospital stay time, dy	11.13 (15.02)	10.89 (15.03)	13.33 (14.72)	0.031
ICU stay time, dy	6.16 (7.41)	5.97 (7.37)	7.91 (7.61)	< 0.001

Continuous variables were presented as mean (SD), categorical variables were presented as numbers (%).

ICU, intensive care unit.

### 3.4. Association of hypocalcemia with in-hospital and ICU mortalities

Hypocalcemia was associated to an increased risk for in-hospital and ICU mortalities in patients with ICH, as shown in Table 3. In the model 1, ICH patients with hypocalcemia had an 83% increased risk of in-hospital mortality (OR = 1.83, 95% CI: 1.31–2.56, *P* = 0.004) and a 115% increased risk of ICU mortality (OR = 2.15, 95% CI: 1.48–3.13, *P* < 0.001). Compared to patients without hypocalcemia, the risk of in-hospital mortality in patients with hypocalcemia increased by 67% (OR = 1.67, 95% CI: 1.09–2.56, *P* = 0.018) in the model 3, and the risk of ICU mortality in patients with hypocalcemia increased by 72% (OR = 1.72, 95% CI: 1.06–2.77, *P* = 0.027).

**Table 3:** Association of hypocalcemia with mortality in critically ill patients with ICH.

	**OR (95%CI)**, ***P*****-value**		
	**Model 1**	**Model 2**	**Model 3**
**In-hospital mortality**			
Non-hypocalcemia	Ref	Ref	Ref
Hypocalcemia	1.83 (1.31, 2.56)	2.17 (1.52,3.08)	1.67 (1.09, 2.56)
	0.004	< 0.001	0.018
**ICU mortality**			
Non-hypocalcemia	Ref	Ref	Ref
Hypocalcemia	2.15 (1.48, 3.13)	2.23 (1.50, 3.31)	1.72 (1.06, 2.77)
	< 0.001	< 0.001	0.027

Model 1: no covariates were adjusted.

Model 2: adjusted for age, gender, race/ethnicity.

Model 3: adjusted for: age, gender, race/ethnicity, region, causes of ICH, body mass index, systolic blood pressure, white blood cell count, hemoglobin, platelet, glutamic-pyruvic transaminase, international normalized ratio, prothrombin time, blood urea nitrogen, creatinine, magnesium, glucose, lactate, sequential organ failure assessment score, first day vasopressor, first day sedative, atrial fibrillation, congestive heart failure.

OR, odds ratio; 95%CI, 95% confidence interval; ICU, intensive care units.

### 3.5. Subgroup analysis

**Figure 2:**
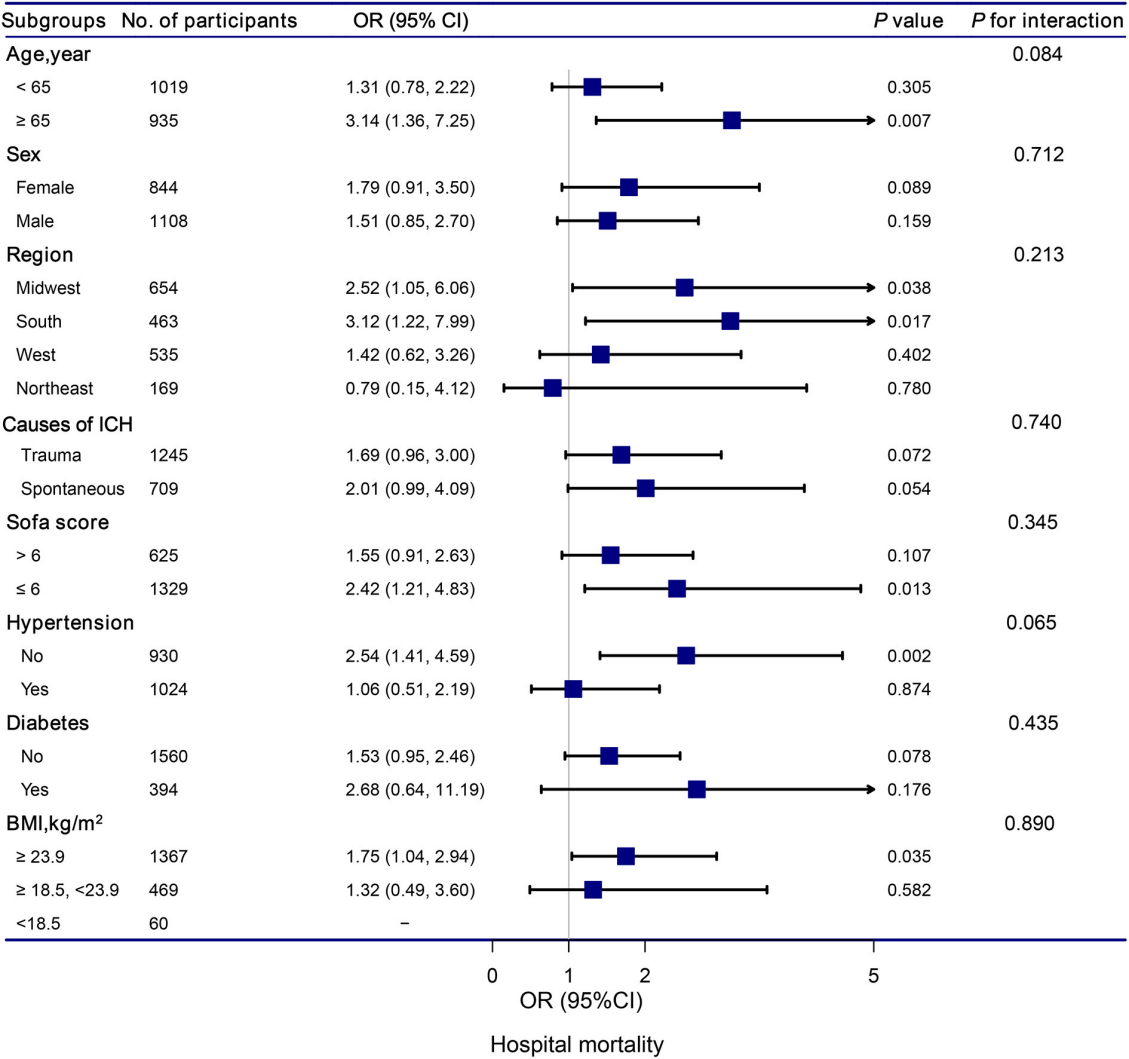
Association between hypocalcemia and hospital mortality according to subgroup. Analyses were adjusted for gender, age, region, race, BMI, systolic blood pressure, diastolic blood pressure, white blood cell count, hemoglobin, platelet, glutamic-pyruvic transaminase, INR, prothrombin time, blood urea nitrogen, creatinine, magnesium, glucose, lactate, SOFA, trauma, first day vasopressor, first day sedative, hypertention, coronary artery disease, atrial fibrillation, congestive heart failure, diabetes, chronic pulmonary disease, and stroke.

**Figure 3:**
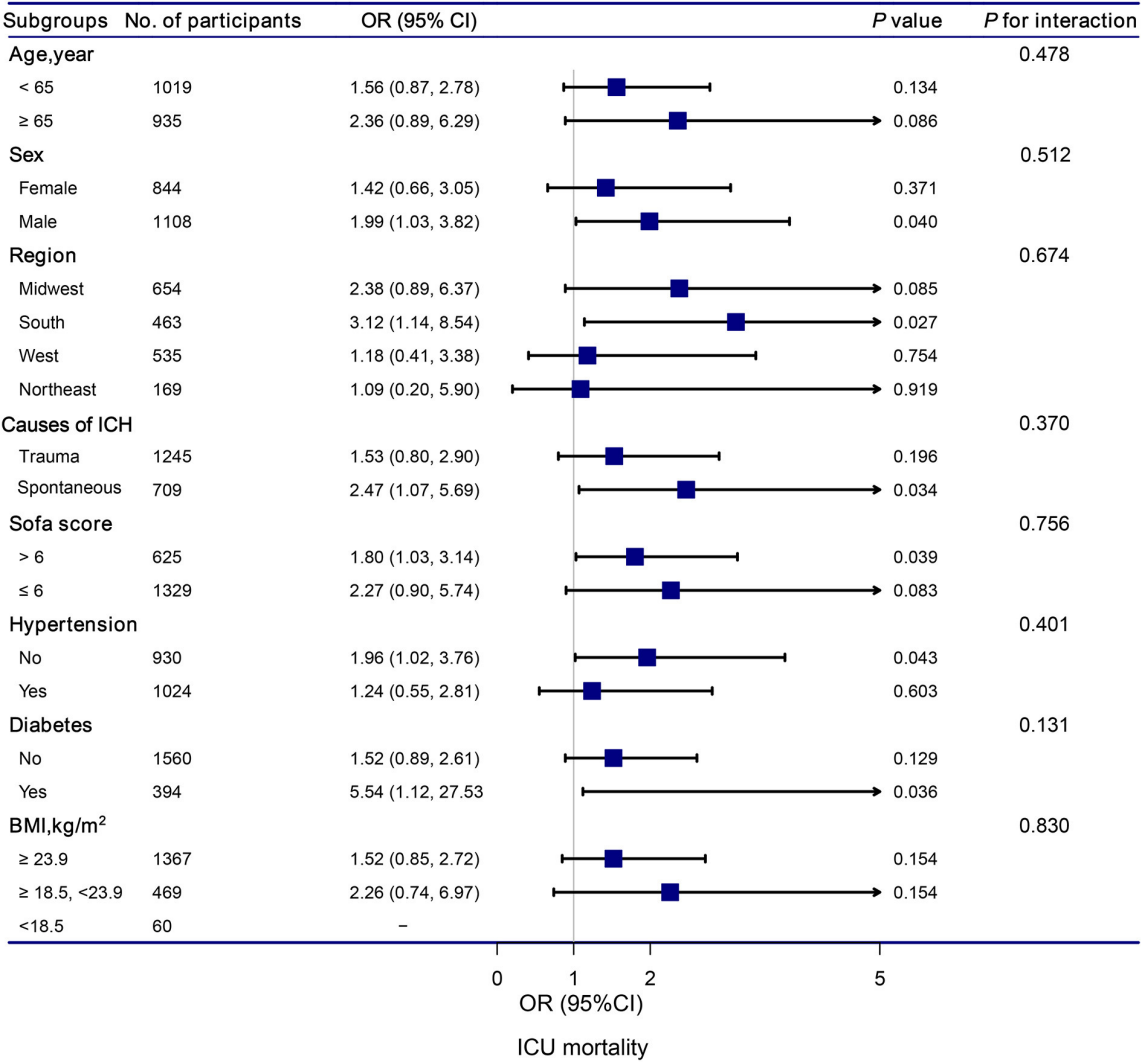
Association between hypocalcemia and ICU mortality according to subgroup. Analyses were adjusted for gender, age, region, race, BMI, mean systolic, mean diastolic, white blood cell count, hemoglobin, platelet, glutamic-pyruvic transaminase, INR, prothrombin time, blood urea nitrogen, creatinine, magnesium, glucose, lactate, SOFA, trauma, first day vasopressor, first day sedative, hypertention, coronary artery disease, atrial fibrillation, congestive heart failure, diabetes, chronic pulmonary disease, and stroke.

The stratification and interaction analyses of the association between hypocalcemia and in-hospital and ICU mortalities were depicted in Figures 2, 3. Subgroup analysis results were in concordance with those of the multivariable logistic regression analysis. The results of the interaction analysis revealed that there were no significant interactions in the subgroups of age, sex, region, causes of ICH, SOFA score, histories of hypertension and diabetes, and BMI.

### 3.6. Kaplan–Meier survival curve

**Figure 4:**
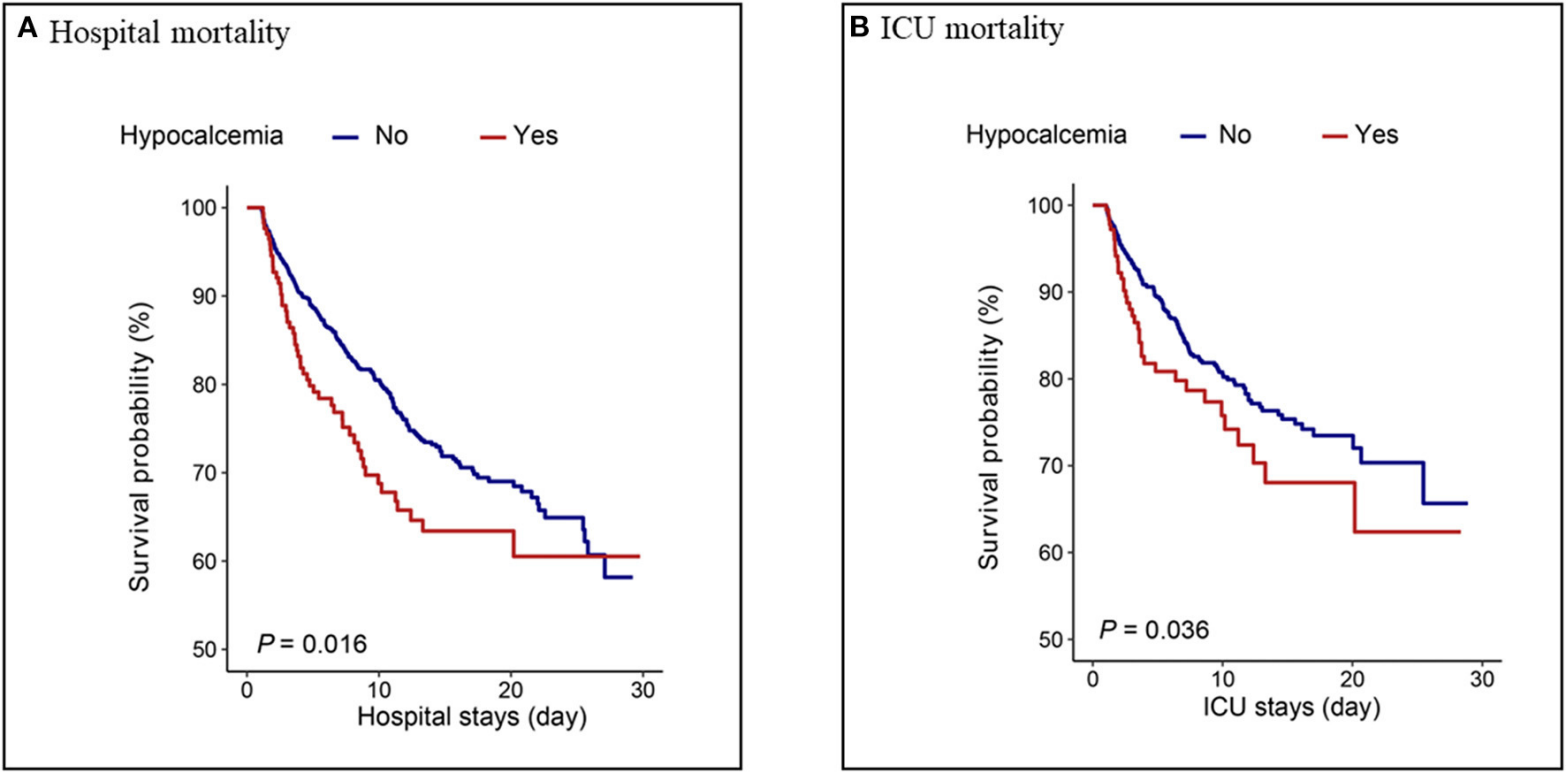
Kaplan–Meier analysis for **(A)** hospital mortality and **(B)** ICU mortality in hypocalcemia and non-hypocalcemia group.

Patients in the hypocalcemia group had a significantly lower survival rate than those without hypocalcemia within 30 days of hospital stay (*P* = 0.016) and 30 days of ICU stay (*P* = 0.036), as shown in Figure 4.

### 3.7. Sensitivity analyses

To test the robustness of the main results, we calculated *E*-values to assess the effect of unmeasured confounding factors. The correlation between hypocalcemia and the risk of ICU mortality was found to be robust, unless the OR of the risk of ICU mortality of an unmeasured confounder was >2.83.

In addition, we performed a sensitivity analysis using data from our own hospital. Patients who were diagnosed with hemorrhagic stroke, subarachnoid hemorrhage, and intraventricular hemorrhage transfered to ICU in our institute were screened retrospectively. A total of 244 patients aged 16–70 years admitted to our institute from January 2010 to October 2022 were analyzed. We attached the results to the Supplementary Tables 1–3 and Supplementary Figure 1. The results of the multivariable logistic regression analysis in the cohort of our institute were in concordance with those in the cohort of eICU-CRD (Supplementary Table 3).

## 4. Discussion

In this retrospective cohort study, we examined the association between hypocalcemia and in-hospital and ICU mortalities in critically ill patients with ICH. We included 1,954 confirmed patients with ICH and divided them into hypocalcemia and non-hypocalcemia groups according to albumin-adjusted total Ca levels. Adjusted for major confounders, our results suggested that hypocalcemia patients with ICH at ICU admission have an increased risk of in-hospital and ICU mortalities. In patients with hypocalcemia, we observed a 67% increased risk of in-hospital mortality and a 72% increased risk of ICU mortality compared to those without hypocalcemia.

In recent years, studies had found that hematoma expansion in patients with spontaneous ICH is related to the serum Ca level at admission (19). A single-center retrospective cohort study from Japan included 273 patients with non-traumatic ICH divided into quartiles based on admission serum Ca levels. After adjusting for other variables, they found that patients in the low serum calcium levels had significantly larger hematoma volumes (18 ml), when compared with that in the higher serum Ca levels group (*P* = 0.025) (20). Another prospective cohort study analyzed 2,103 patients with primary ICH (12). They reported that patients with hypocalcemia had a higher baseline hematoma volume than patients without hypocalcemia. In a subgroup of 1,309 patients, a higher blood Ca concentration was associated to a decreased risk of ICH expansion (OR = 0.55; 95% CI, 0.35–0.86; *P* = 0.01). According to another study, low ionized Ca levels were associated with a poor prognosis following early hematoma expansion in 111 patients with hypertensive ICH (21).

The causal relationship and precise mechanism between hypocalcemia and mortality are unclear. According to previous studies, the mechanism might be as follows: Firstly, Ca plays a crucial role in the coagulation cascade (22). Therefore, hypocalcemia patients with ICH may have impaired hemostasis, promoting ICH progression, and increasing the risk of mortality. Secondly, serum Ca levels are correlated with PLT function and several steps of PLT aggregation (23, 24). Hypocalcemia may be accompanied by PLT dysfunction and a poor prognosis. Thirdly, activation of the systemic immune response after ICH leads to PTH-vitamin D axis dysfunction, low serum Ca levels, or hypocalcemia (25). Hypocalcemia is common in patients requiring ICU admission and is associated to increased mortality (26). The specific mechanism requires further research.

Subgroup analyses based on the causes of ICH revealed that traumatic ICH patients with hypocalcemia had a 147% elevated risk of ICU mortality (OR = 2.47, 95% CI: 1.07–5.69, *P* = 0.034). Vinas-Rios et al. (27) conducted an ambispective comparative case-control study and suggested that traumatic hypocalcemia patients with ICH had an increased risk of mortality (OR = 5.2; 95% CI: 4.48–6.032). Our results were consistent with their findings. Patients with traumatic ICH have a higher incidence of hypocalcemia. The mechanism may be as follows: in patients with traumatic ICH, the decrease in serum Ca is related to bonding with the complex Calcium/calmodulin-dependent protein kinases II and lactic acid (28, 29). Transmembrane Ca input after traumatic cell membrane deformation leads to acute elevation of intracellular Ca levels, which can cause neurological disorders, along with death (30).

Subgroup analyses also revealed that hypocalcemia patients with diabetes had a significantly increased risk of ICU mortality compared to those without hypocalcemia. ICH is a subtype of stroke associated to higher mortality (31), particularly in the population with diabetes mellitus (DM) (32). The pathophysiological processes underlying ICH-induced brain damage are highly influenced by the presence of DM (33). DM promotes massive blood-brain barrier destruction after ICH by affecting pericytes, endothelial cells, and tight junction proteins, leading to vasogenic edema and hematoma expansion (34–36). Future studies should aim to provide a better understanding of pathophysiological changes in patients with ICH and DM. These patients deserve more attention, and it is important to develop an appropriate treatment strategy for patients with ICH and DM.

In this study, we also found that ICH complicated by hypocalcemia was not correlated with the underlying disease but was correlated with the severity of the condition since patients with ICH, complicated by hypocalcemia, had higher SOFA scores. Previous studies have clarified that for organs other than the liver, the SOFA score of each organ has a significant correlation with in-hospital mortality during the patient's ICU period (37, 38); in particular, the neurological score has the greatest impact on prognosis (38). This is consistent with the conclusions of our study.

The present study has several strengths. Firstly, this was the first study based on the association between hypocalcemia and hospital mortality in patients with ICH using multicenter ICU data from the United States. Secondly, a relatively large sample was used in this study; therefore, subgroup analysis could be conducted. The results were stable in each subgroup, and no interactions were found. However, this study also has several limitations. First, our analyses are retrospective and based on observational studies; therefore, they cannot establish a causal association between hypocalcemia and hospital mortality in patients with ICH. Second, although the E-value analysis suggested that certain confounding factors were unlikely to effect the risk of ICU mortality, the possibility of confounding effects of incomplete adjustment for some ICH risk factors such as hematoma volume, ICH score and oral anticoagulation cannot be excluded. Third, since the cohort participants were ICU patients, our conclusions may not generalize to other populations.

## 5. Conclusions

In this multicenter cohort, we found that hypocalcemia was associated with an elevated risk of in-hospital and ICU mortalities in critically ill patients with ICH. Further randomized clinical trials and prospective studies are needed to validate our findings.

## Data availability statement

The data analyzed in this study was obtained from the e-Intensive Care Unit Collaborative Research Database (eICU-CRD), the following licenses/restrictions apply: To access the files, users must be a credentialed user, complete the required training (CITI Data or Specimens Only Research) and sign the data use agreement for the project. Requests to access these datasets should be directed at PhysioNet, https://physionet.org/, https://doi.org/10.13026/C2WM1R.

## Ethics statement

Ethical review and approval was not required for the study on human participants in accordance with the local legislation and institutional requirements. Written informed consent from the patients/participants or patients/participants' legal guardian/next of kin was not required to participate in this study in accordance with the national legislation and the institutional requirements.

## Author contributions

FGu and WZ: writing—original draft preparation. XL, YZ, XJ, GC, XD, and HP: validation. FGa and HW: writing—review and editing. All authors contributed to the article and approved the submitted version.

## Supplementary material

Supplementary Figure 1 Kaplan–Meier analysis for hospital mortality in hypocalcemia and non-hypocalcemia group.

Supplementary Table 1 Characteristics of study participants.

Supplementary Table 2 The outcomes of patients in hypocalcemia and non-hypocalcemia group.

Supplementary Table 3 Association of hypocalcemia with hospital mortality in critically ill patients with ICH.
