# Supplementary figures and images for: Association of hypocalcemia with in-hospital mortality in critically ill patients with intracerebral hemorrhage: A retrospective cohort study
Source: Front Neurol. 2023 Jan 9;13:1054098. doi: 10.3389/fneur.2022.1054098 (PMC9868589; doi:10.3389/fneur.2022.1054098)

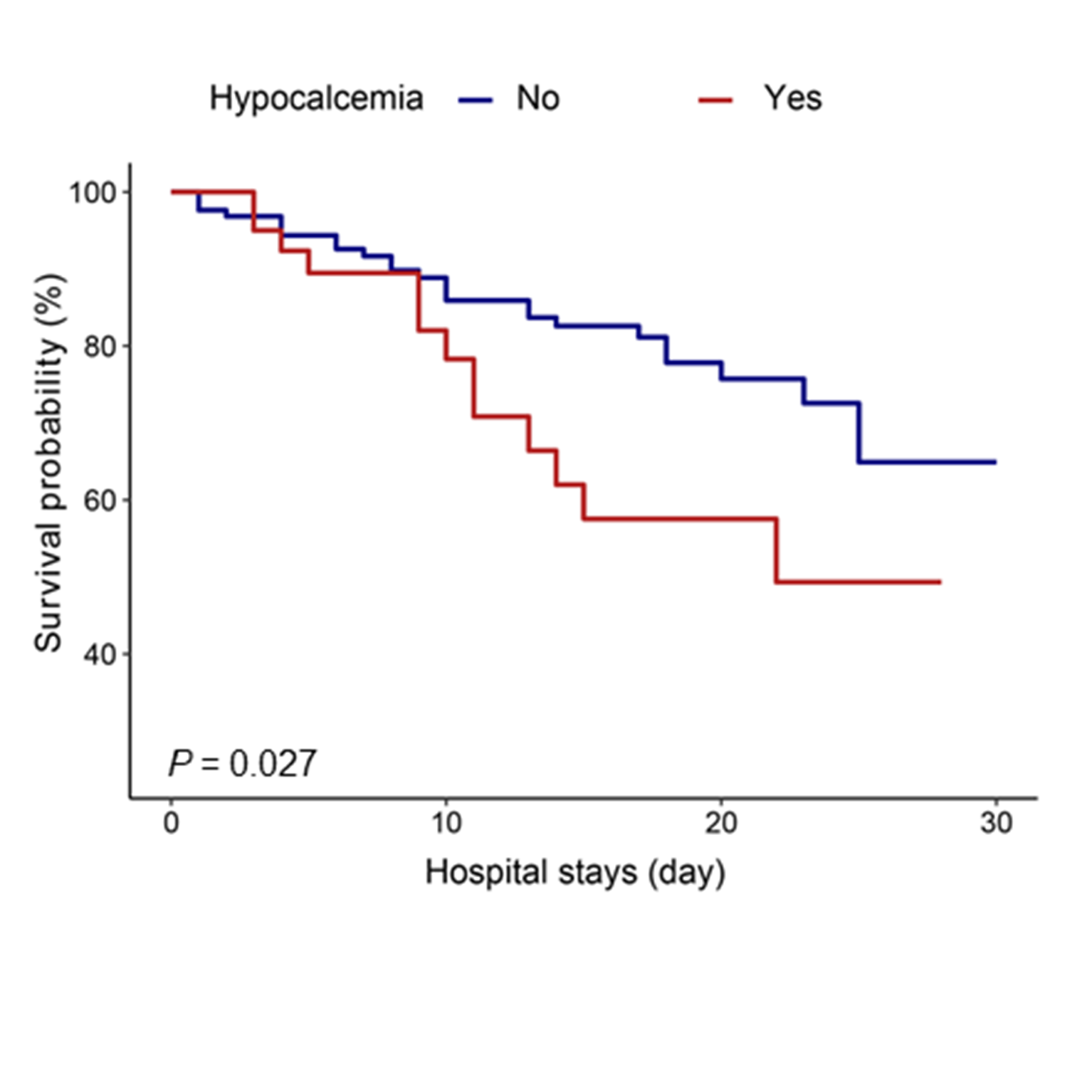

Supplement: Supplementary Figure 1 — Kaplan–Meier analysis for hospital mortality in hypocalcemia and non-hypocalcemia group. [file Image_1.TIF]
