# Supplementary material for: Association of hypocalcemia with in-hospital mortality in critically ill patients with intracerebral hemorrhage: A retrospective cohort study
Source: Front Neurol. 2023 Jan 9;13:1054098. doi: 10.3389/fneur.2022.1054098 (PMC9868589; doi:10.3389/fneur.2022.1054098)
Supplement: Supplementary Table 1 — Characteristics of study participants. [file Table_1.DOCX]

eTable 1. Characteristics of study participants

|  |  | Albumin-corrected total calcium, mg/dL | | |
| --- | --- | --- | --- | --- |
|  |  | Non-hypocalcemia | Hypocalcemia |  |
| Characteristics | Total | ≥8.4 | <8.4 *P-*value | |
| N | 244 | 184 | 60 |  |
| Age, years | 55.58 (10.99) | 55.42 (11.22) | 56.07 (10.35) | 0.693 |
| Gender, N (%) |  |  |  | 0.837 |
| Male | 160 (65.57) | 120 (65.22) | 40 (66.67) |  |
| GCS score | 5.25 (2.74) | 5.28 (2.87) | 5.17 (2.38) | 0.857 |
| Physical examination |  |  |  |  |
| BMI, kg/m^2^ | 24.77 (4.43) | 25.33 (4.52) | 21.84 (2.38) | 0.029 |
| SBP, mmhg | 134.71 (28.05) | 134.53 (27.50) | 135.29 (30.02) | 0.860 |
| DBP, mmhg | 81.70 (17.83) | 81.58 (18.70) | 82.07 (14.86) | 0.859 |
| Laboratory data |  |  |  |  |
| Glucose, mmol/L | 8.26 (3.37) | 8.28 (3.55) | 8.21 (2.80) | 0.893 |
| WBC count, ×10^3^/μL | 13.72 (6.02) | 13.66 (6.25) | 13.91 (5.28) | 0.784 |
| Hb, g/mL | 116.00 (26.44) | 116.64 (25.37) | 114.07 (29.56) | 0.515 |
| PLT, ×10^3^/uL | 188.85 (90.30) | 196.56 (91.63) | 165.20 (82.36) | 0.019 |
| INR | 1.14 (0.22) | 1.13 (0.21) | 1.16 (0.27) | 0.455 |
| PT | 12.96 (2.68) | 12.87 (2.58) | 13.22 (2.96) | 0.388 |
| BUN, mmol/L | 6.36 (5.04) | 6.28 (4.57) | 6.63 (6.29) | 0.637 |
| Creatinine, µmol/L | 101.13 (128.31) | 101.46 (136.61) | 100.14 (100.34) | 0.945 |
| Lactate, mmol/L | 2.25 (2.29) | 2.18 (2.43) | 2.54 (1.66) | 0.440 |
| ALT, U/L | 46.74 (93.83) | 39.86 (62.89) | 67.83 (152.93) | 0.045 |
| Magnesium, mmol/L | 0.84 (0.16) | 0.85 (0.15) | 0.81 (0.19) | 0.132 |
| Comorbidities |  |  |  |  |
| Hypertention, N (%) | 106 (44.17) | 87 (48.07) | 19 (32.20) | 0.033 |
| Diabetes, N (%) | 20 (8.33) | 14 (7.73) | 6 (10.17) | 0.557 |
| Causes of ICH, N (%) |  |  |  |  |
| Trauma | 48 (19.67) | 31 (16.85) | 17 (28.33) | 0.052 |
| Spontaneous | 196 (80.33) | 153 (83.15) | 43 (71.67) |  |
| First day sedative, N (%) 140 (57.38) 106 (57.61) | | | 34 (56.67) | 0.898 |
| First day vasopressor, N (%) 74 (30.33) 53 (28.80) | | | 21 (35.00) | 0.365 |

Note: Continuous variables were presented as mean (SD), calculated by linear regression model. Categorical variables were presented as numbers (%), calculated by chi-square test.

Abbreviations: SD, standard deviation; BMI, Body Mass Index; SBP, systolic blood pressure; DBP, diastolic blood pressure; WBC, white blood cell; PLT, platelet; INR, International normalized ratio; PT, prothrombin time; BUN, Blood urea nitrogen; ALT, alanine aminotransferase; Hb, hemoglobin; GCS, Glasgow coma scale.
