# Supplementary material for: Association of hypocalcemia with in-hospital mortality in critically ill patients with intracerebral hemorrhage: A retrospective cohort study
Source: Front Neurol. 2023 Jan 9;13:1054098. doi: 10.3389/fneur.2022.1054098 (PMC9868589; doi:10.3389/fneur.2022.1054098)
Supplement: Supplementary Table 2 — The outcomes of patients in hypocalcemia and non-hypocalcemia group. [file Table_2.DOCX]

eTable 2. The outcomes of patients in hypocalcemia and non-hypocalcemia group

|  | Albumin-corrected total calcium, mg/dL | | | | |
| --- | --- | --- | --- | --- | --- |
|  |  | | Non-hypocalcemia Hypocalcemia | |  |
| N | Total | | ≥8.4 | <8.4 | P-value |
|  | | 244 | 184 | 60 |  |
| In-hospital mortality, N (%) | | 42 (17.21） | 26 (14.13) | 16 (26.67) | 0.025 |
| Hospital stay time, d | | 30.59 (36.20) | 31.79 (37.39） | 26.95 (32.30) | 0.370 |

Note: Continuous variables were presented as mean (SD), Categorical variables were presented as numbers (%). For continuous variable, Kruskal Wallis rank sum test was used. If the counting variable has a theoretical number <10, it is obtained by Fisher^,^s exact probability test.
