# Supplementary material for: Association of hypocalcemia with in-hospital mortality in critically ill patients with intracerebral hemorrhage: A retrospective cohort study
Source: Front Neurol. 2023 Jan 9;13:1054098. doi: 10.3389/fneur.2022.1054098 (PMC9868589; doi:10.3389/fneur.2022.1054098)
Supplement: Supplementary Table 3 — Association of hypocalcemia with hospital mortality in critically ill patients with ICH. [file Table_3.DOCX]

eTable 3. Association of hypocalcemia with hospital mortality in critically ill patients with ICH

|  | OR (95%CI), *P*-value | | |
| --- | --- | --- | --- |
|  | Model 1 | Model 2 | Model 3 |
| Hospital mortality | |  |  |
| Non-hypocalcemia Ref Ref Ref | | | |
| Hypocalcemia | 2.21 (1.09, 4.48) 0.028 | 2.19 (1.078, 4.45) 0.031 | 2.61 (1.05, 6.49) 0.040 |

Model 1: no covariates were adjusted.

Model 2: adjusted for age, gender.

Model 3: adjusted for: age, gender, causes of ICH, Glasgow coma scale score, body mass index, systolic blood pressure, diastolic blood pressure, white blood cell count, hemoglobin, platelet, alanine aminotransferase, international normalized ratio, prothrombin time, blood urea nitrogen, creatinine, magnesium, glucose, lactate, first day vasopressor, first day sedative, hypertension, diabetes.

Abbreviation: OR, odds ratio; 95%CI, 95% confidence interval; ICU, intensive care units.
